# Distribution of disease-causing germline mutations in coiled-coils implies an important role of their N-terminal region

**DOI:** 10.1038/s41598-020-74354-9

**Published:** 2020-10-15

**Authors:** Zsofia E. Kalman, Bálint Mészáros, Zoltán Gáspári, Laszlo Dobson

**Affiliations:** 1 Faculty of Information Technology and Bionics, Pázmány Péter Catholic University, Práter u. 50/A, 1083 Budapest, Hungary; 2 3in-PPCU Research Group, 2500 Esztergom, Hungary; 3 Structural and Computational Biology Unit, European Molecular Biology Laboratory, Meyerhofstraße 1, 69117 Heidelberg, Germany; 4 Research Centre for Natural Sciences, Magyar Tudósok Körútja 2, 1117 Budapest, Hungary

**Keywords:** Protein sequence analyses, Protein structure predictions, Protein folding, Proteomics, Diseases

## Abstract

Next-generation sequencing resulted in the identification of a huge number of naturally occurring variations in human proteins. The correct interpretation of the functional effects of these variations necessitates the understanding of how they modulate protein structure. Coiled-coils are α-helical structures responsible for a diverse range of functions, but most importantly, they facilitate the structural organization of macromolecular scaffolds via oligomerization. In this study, we analyzed a comprehensive set of disease-associated germline mutations in coiled-coil structures. Our results suggest an important role of residues near the N-terminal part of coiled-coil regions, possibly critical for superhelix assembly and folding in some cases. We also show that coiled-coils of different oligomerization states exhibit characteristically distinct patterns of disease-causing mutations. Our study provides structural and functional explanations on how disease emerges through the mutation of these structural motifs.

## Introduction

Advances in sequencing resulted in the identification of a huge number of different Single Nucleotide Variations (SNVs) of various genomic positions among individuals in healthy and disease states. Variations can impact proteins on several levels, ranging from polymorphisms (PMs) with negligible effect on fitness to lethal mutations through increasingly strong phenotypes. According to their origin, disease-causing genetic alterations can be broadly categorized into either somatic or germline mutations. Somatic mutations, most notably responsible for tumorigenesis, are confined to the cell they originated in and its daughter cells. In accord, their phenotypic change can be extreme with abolishing cell-cycle control, escaping apoptosis and achieving cellular immortality. In contrast, disease-associated germline mutations (DMs) persist in all cells of the organism and are transmitted from generation to generation. Thus, DMs cause relatively weak changes in phenotypes, yet they still have a noticeable negative impact on the quality of life.

Coiled-coils are oligomeric helical structural units in proteins connected to a wide range of functions. Several coiled coil proteins have been shown to have catalytic activity^1^ or undergo oligomerization, yet their most common functions arise directly from their structure: they are molecular spacers separating or connecting domains^2^. They can bridge large distances and connect proteins at different sides of large supramolecular structures, like the postsynaptic density where the Homer coiled coil serves as a direct connection between the plasma membrane and intracellular proteins through EVH1 domains binding to various scaffolding partners^3^.

Coiled-coils are α-helical domains consisting of two or more helices packed together in a specific knobs-into-holes manner^4^, with interhelical interactions playing a dominant role in folding^5^. Coiled-coils can have parallel or antiparallel arrangement, and they can be formed by intrachain interaction of the same subunit, or by interchain bonds between distinct polypeptide chains^6^. Regardless of their oligomerization state, the main forces driving their interactions are the formation of hydrophobic contacts at their inside, often supported by electrostatic interactions aiding the stability from the outside^7^. Coiled-coil residues can be classified into register positions according to their role in complex formation with the opposing helices: the most common repeat pattern is a heptad (‘abcdefg’), with ‘a’ and ‘d’ positions being responsible for hydrophobic interactions, while ‘e’ and ‘g’ positions contain (oppositely) charged residues^8^. Notably, other variants (e.g., hendecads are 11 residue repeats) were also discovered^9^. Folding studies of selected coiled-coils indicated the importance of a specific segment, the trigger sequence, that is required for initiating the proper interaction between the helices^10^. However, it is not yet entirely clear whether specific sequence patterns are required for assembly, or the accumulation of interaction promoting residues at critical positions generally aid coiled-coil formation.

In recent decades many studies addressed how DMs perturb protein structure. The majority of frequently occurring structural elements (e.g., transmembrane^11^ and intrinsically disordered protein regions^12^), as well as various structurally distinct functional regions (e.g., protein–protein interfaces, buried domains^13^) were analyzed in detail. However, coiled-coils are a largely understudied class in this respect with only individual cases discussed. To our knowledge, only one large-scale study has been published, highlighting the critical role of register positions and pointing out mutations frequently associated with pleiotropy^14^. In this study we integrate multiple prediction algorithms and structural information for an in-depth analysis to assess how non-synonymous disease-associated germline mutations affect coiled-coil structures and thereby their functions. Our work revealed that disrupting hydrophobic and electrostatic interactions impairs coiled-coil structure and disease-associated mutations accumulate near the N-terminal of coiled-coil regions. We also showed that even if their destabilizing effect is small, DMs are enriched in antiparallel homodimer coiled-coils. On one hand, understanding how these variations modulate the structure and function of proteins may improve prediction algorithms. On the other hand, the rational coiled-coil design can be achieved through a detailed understanding of the sequence-structure relationship^15^. However, a missing piece of the puzzle is how DMs perturb coiled-coil structures.

## Results

### DMs are depleted in coiled-coil regions and they are most often associated with central nervous system diseases

To obtain an overall picture of how disease-associated mutations (DM) and coiled-coils are related, we determined the relative frequency of DMs and PMs. We also calculated how proteins having coiled-coil regions are affected. We found DMs are less frequent in coiled-coils producing a 0.56 mean odds ratio, however coiled-coil containing proteins gather nearly the same amount of DMs as other proteins (Supplementary Material, Supplementary Fig. 1). Most coiled-coils (~ 95%) do not contain any variation, and the majority of variations occupy the coiled-coil segment alone. There is a non-significant trend showing a slight increase in the ratio of coiled-coil regions with multiple DMs compared to PMs (Supplementary Fig. 2).

**Figure 1:**
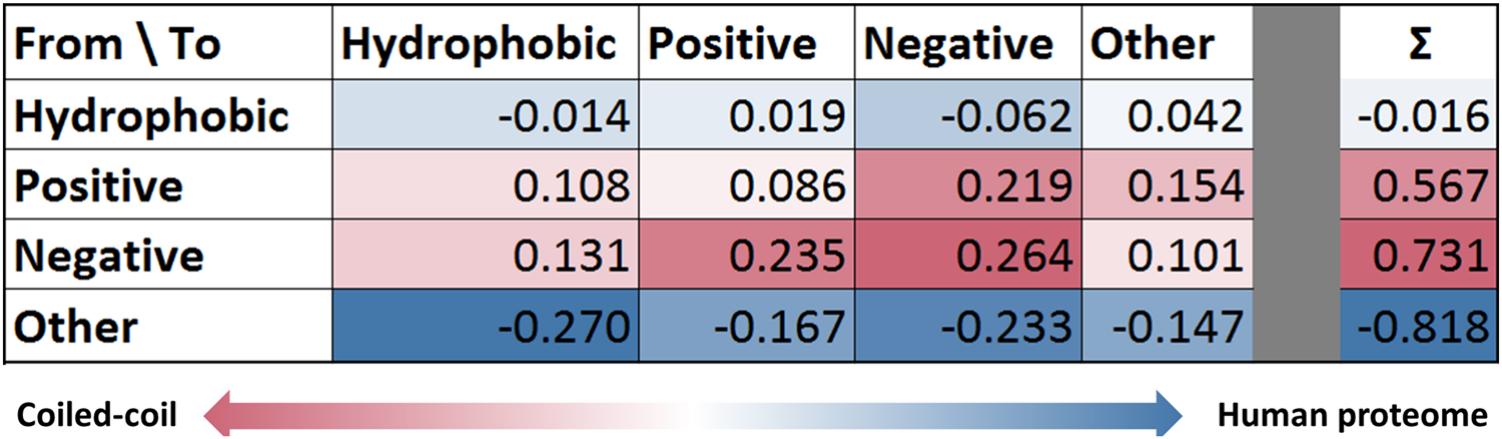
Amino acid changes in coiled-coils. (Left) Residue change preferences by DMs in the proteome (negative values, also marked with the shades of blue) and in coiled-coil regions (positive values, also marked with the shades of red). Values show the logarithm of ratio of DMs in coiled-coils and in the proteome that change the given residues types. (Right) Targeted residue type preferences by DMs in the proteome (negative values) and in coiled-coil regions (positive values).

**Figure 2:**
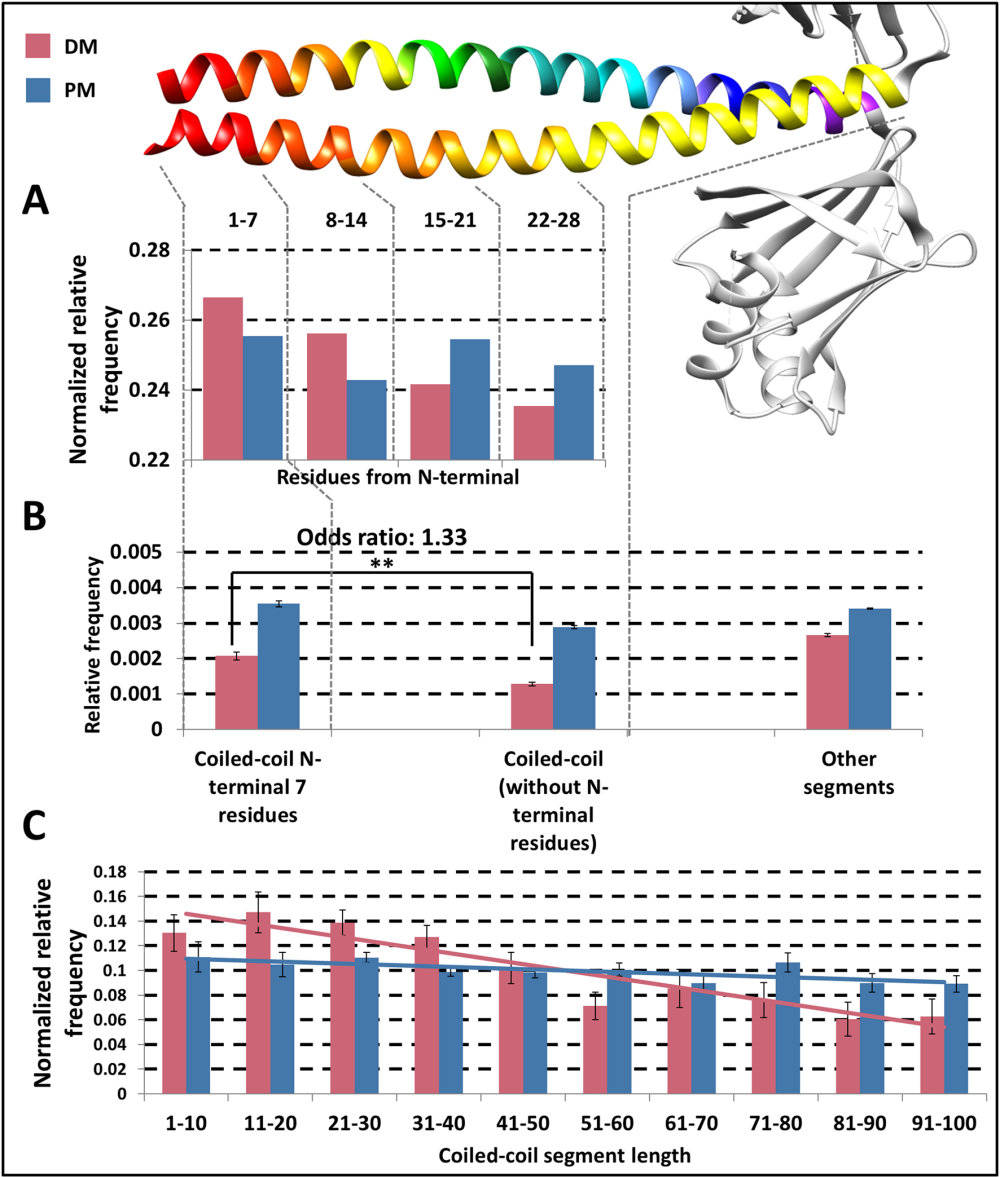
Variations along coiled-coil segments. Distribution of variations in the sequence. (**A**) X-axis shows the coverage of the N-terminal of coiled-coils. (**B**) Relative frequency of coiled-coil residues targeted at N-terminal of the coiled-coil, other coiled-coil residues and other segments of proteins, respectively. (**C**) Distributions of variations in coiled-coils with different lengths (linear trend lines were aligned to the data). Red: DMs; blue: PMs.

To reveal disease groups that are most often associated with DMs falling into coiled-coil regions, we calculated the number of occurrences of each disease category using DiseaseOntology. According to our analysis, the most enriched disease terms are skin diseases, muscular diseases, carbohydrate metabolic diseases, and central nervous system diseases (Supplementary Material, Supplementary Fig. 3).

**Figure 3:**
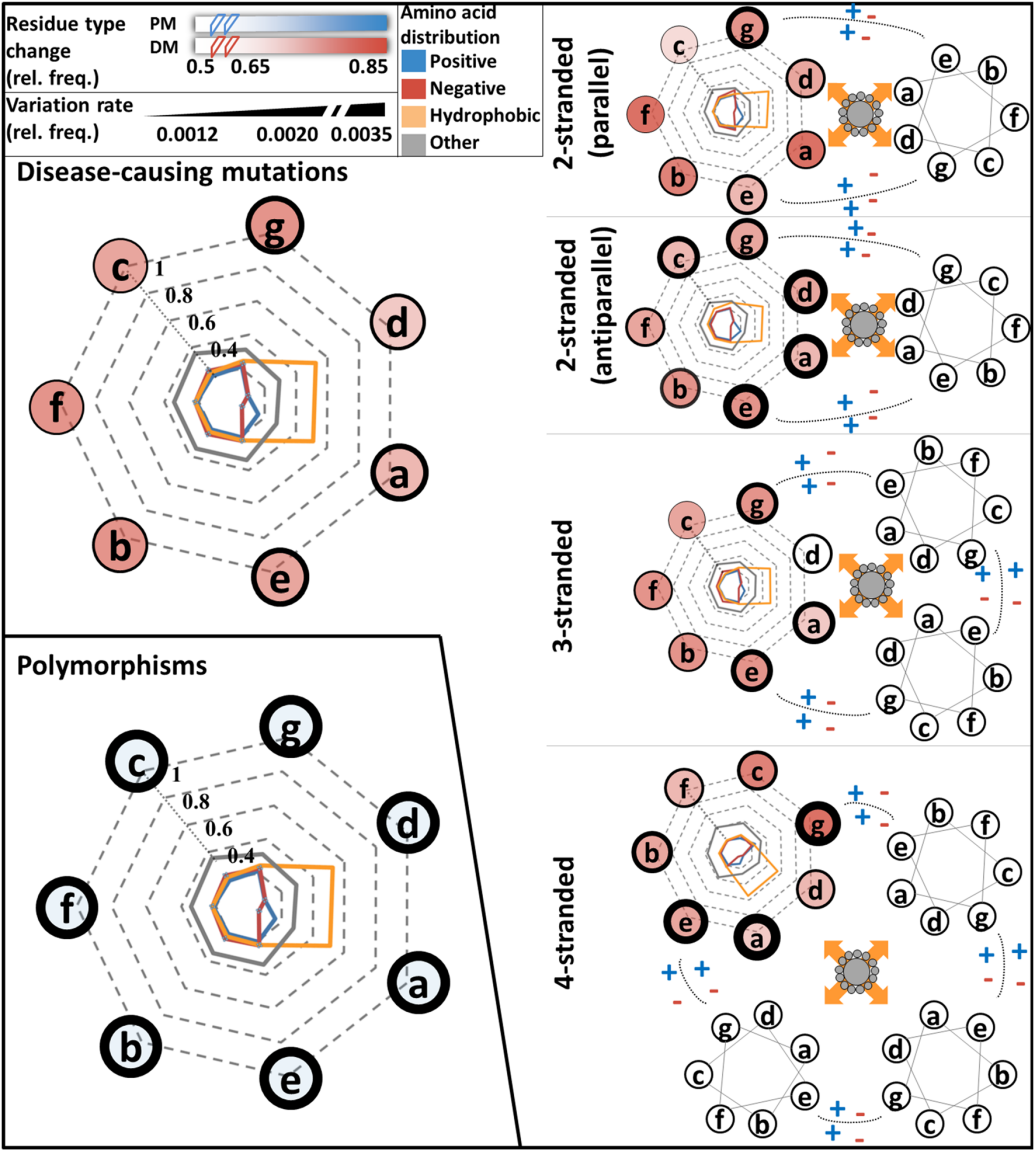
Distribution of variations in coiled-coils. Amino acids were grouped according to their physico-chemical properties (positive: HKR, negative: DE, small hydrophobic: AILMV, other: CFGNPQSTWY). Radars represent the amino acid distributions in different positions. Line thickness around positions is proportional to variation frequency, showing the mean of relative frequencies derived from various predictors. The opacity of positions is proportional to the rate of variations changing the physico-chemical features of the targeted residue. Left: DMs and PMs in coiled-coils. Right: DMs in coiled-coils with different oligomerization states.

### Coiled-coils are often perturbed by DMs affecting charged residues

The main driving force of protein domain folding and stability is achieved through hydrophobic interactions. Coiled-coils are special structural units where the balanced contribution of hydrophobic interactions and electrostatic interactions aid the stability together. This is reflected by the different amino acid preferences of the different positions in the heptad repeat unit, corresponding to the distinct spatial position and role of these within the superhelical structure. To assess how residues are affected by variations, we grouped amino acids based on their basic physico-chemical features (positive: HKR, negative: DE, hydrophobic: AILMV, other: CFGNPQSTWY), then we calculated the log ratio of the substitutions observed in DMs.

Figure 1 shows preferred residue type changes in coiled-coils relative to other non coiled-coil regions of the proteome. We calculated the relative frequency of amino acid substitutions in the coiled-coil regions, and in the proteome, then calculated the log ratio of substitution frequencies. According to our results hydrophobic residues are targeted in similar proportions. However, in the case of coiled-coils, charged residues aiding electrostatic interactions are much more frequently affected by DMs (Fig. 1, right).

In contrast, several residue types indispensable for stable domain structure (e.g., cysteines forming disulfide bridges) do not influence coiled-coil formation, thus their replacement does not cause stability problems (Fig. 1, left, for more details see Supplementary Fig. 4). The most prevalent changes in coiled-coil regions by DMs are replacements by oppositely charged residues. Interestingly, the negatively charged Glu and Asp are generally not interchangeable residues in coiled-coils, in contrast to the positively charged residues Lys and Arg. In coiled-coils DMs most likely target A, E, I, K, L, M, N and Q residues, as opposed to C, G and P residues being more often targeted in other proteins in the proteome (Supplementary Fig. 4). Both the non-redundant and the full human proteome show similar trends (Supplementary Table 12).

**Figure 4:**
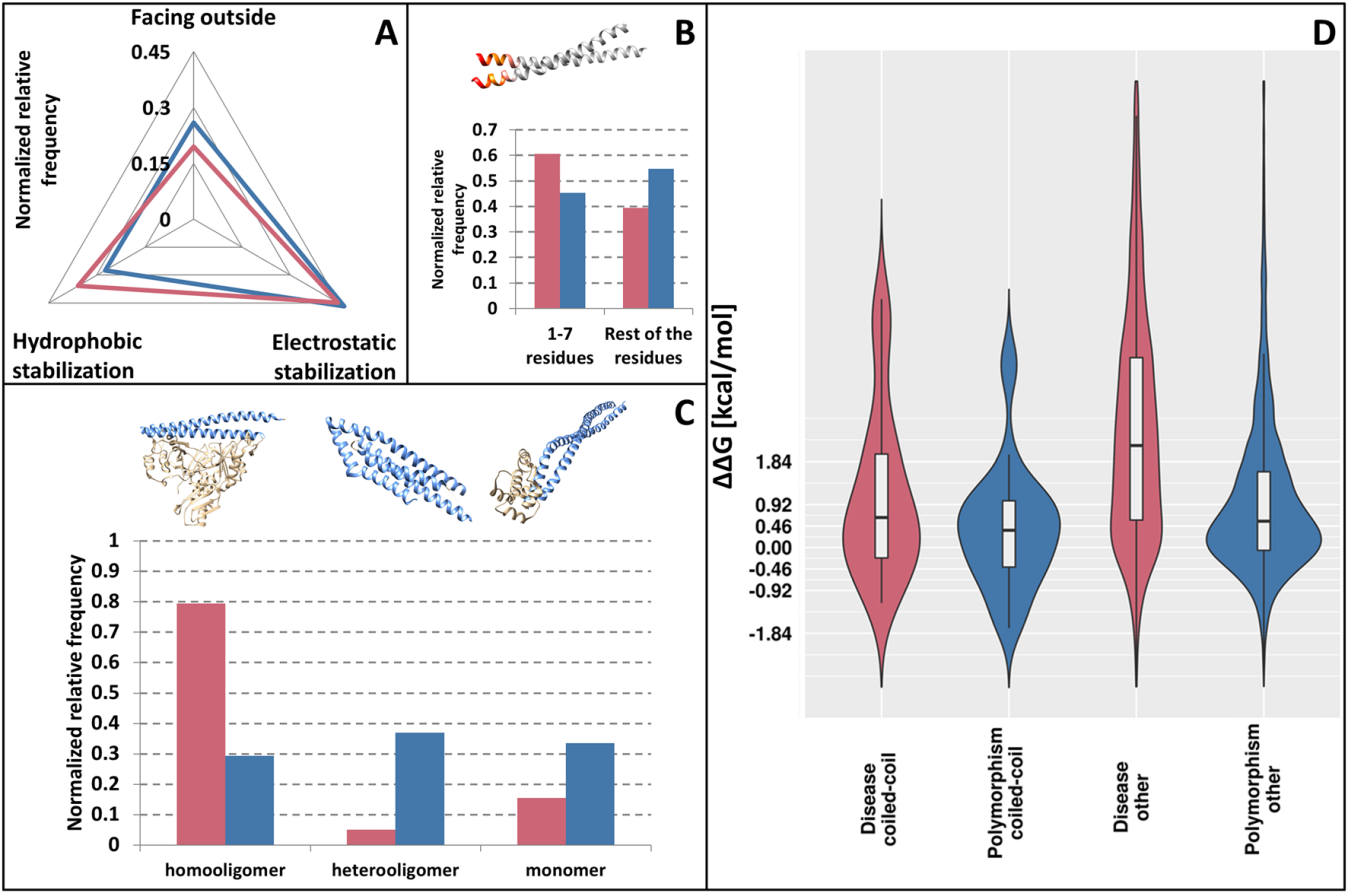
Distribution of variation on human non-redundant PDB structures. (**A**) Distribution of variations based on the register position types. (**B**) Distribution of variations in the N-terminal seven residues and in other segments of coiled-coils. (**C**) Distribution of variations according to the oligomerization state of structures. (**D**) Energy change distributions in coiled-coils (right) and in other proteins from the human proteome (left). Red: DMs; blue: PMs.

### DMs accumulate at the N-terminal region of coiled-coils

We investigated the distribution of variations in coiled-coils, considering their coverage, abundance in the N-terminal region, and coiled-coil length. We divided coiled-coil regions into five equal parts, and calculated the proportion of variations in these parts. Although the first half of the sequences contain slightly more DMs, the difference is not significant compared to PMs (Supplementary Fig. 5).

**Figure 5:**
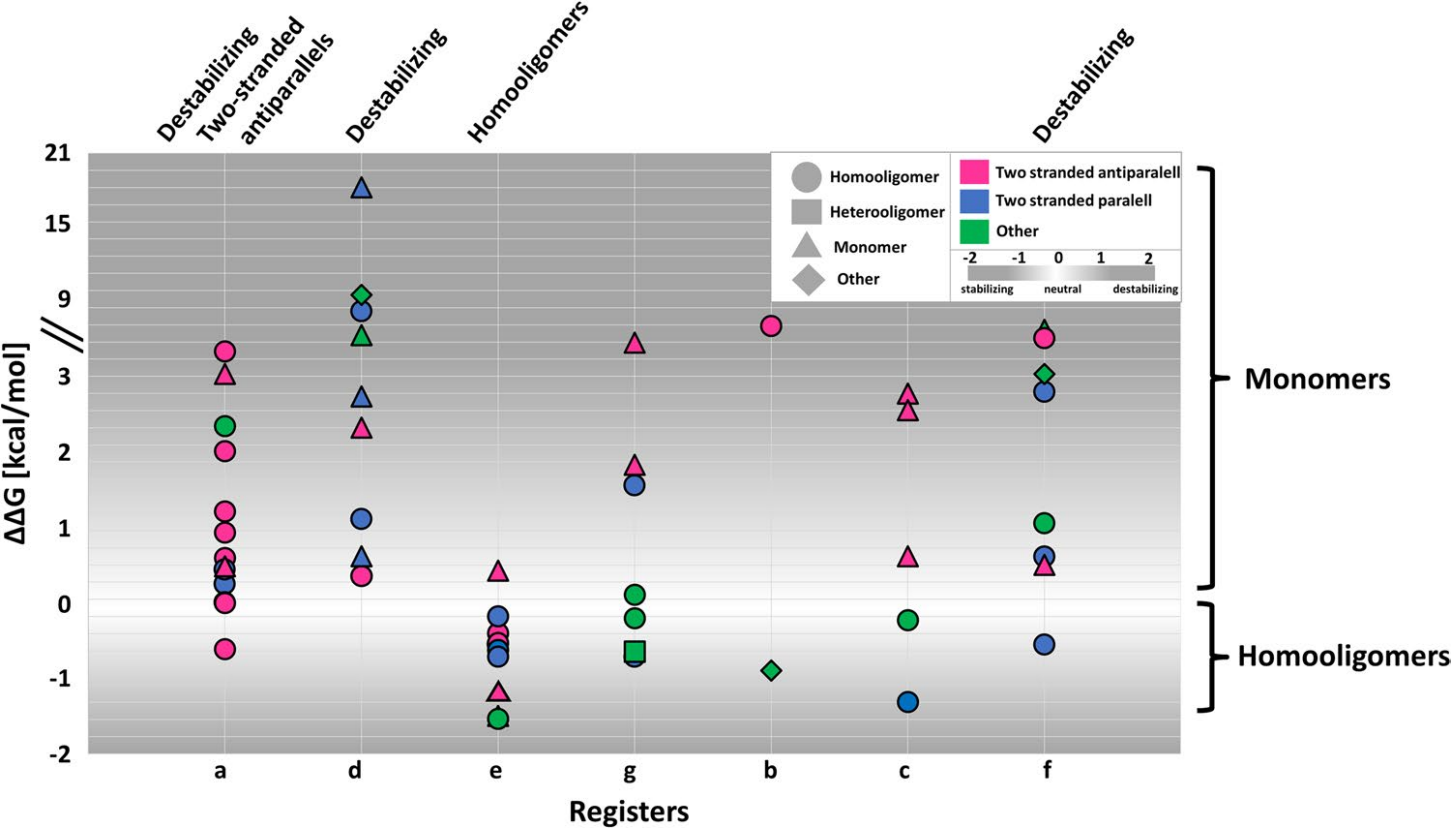
Distribution of DMs with respect to various structural features. Y-axis shows the calculated energy change caused by mutations. X-axis shows the register position DMs fall into. Colors and shapes highlight further properties (see legend).

To reduce bias originating from the varied length of coiled-coils, we performed the enrichment calculation considering only the first 28 residues of all coiled-coils, this time by dividing sequences into four equal parts, i.e., using seven residue bins—keeping in mind that predictors were optimized for heptad repeats (Fig. 2A). Using this approach, the accumulation of DMs at the N-terminal became visible, showing a monotonous decline of DMs towards the C-terminal, however we could not confirm that this trend is significant.

To demonstrate that the first seven residues of coiled-coils contain significantly more DMs compared to the rest of the coiled-coil regions, we counted the number of DMs and PMs in the first seven residues of coiled-coils and in their succeeding part. The result is significant (*χ*^2^ test, *p* < 0.01), and the odds ratio between DMs and PMs is 1.33 (Fig. 2B). This result is confirmed by all predictors independently (Supplementary Fig. 6) and on each dataset (Supplementary Fig. 7). To eliminate possible bias caused by shorter coiled-coil segments, we also shuffled the position of variations inside each protein, and calculated the same statistics. Using this approach no abundance is visible at the N-terminal, the variations randomly distributed along the sequence without any significant accumulation (*p* > 0.01 with all predictors) (Supplementary Table 7).

**Figure 6:**
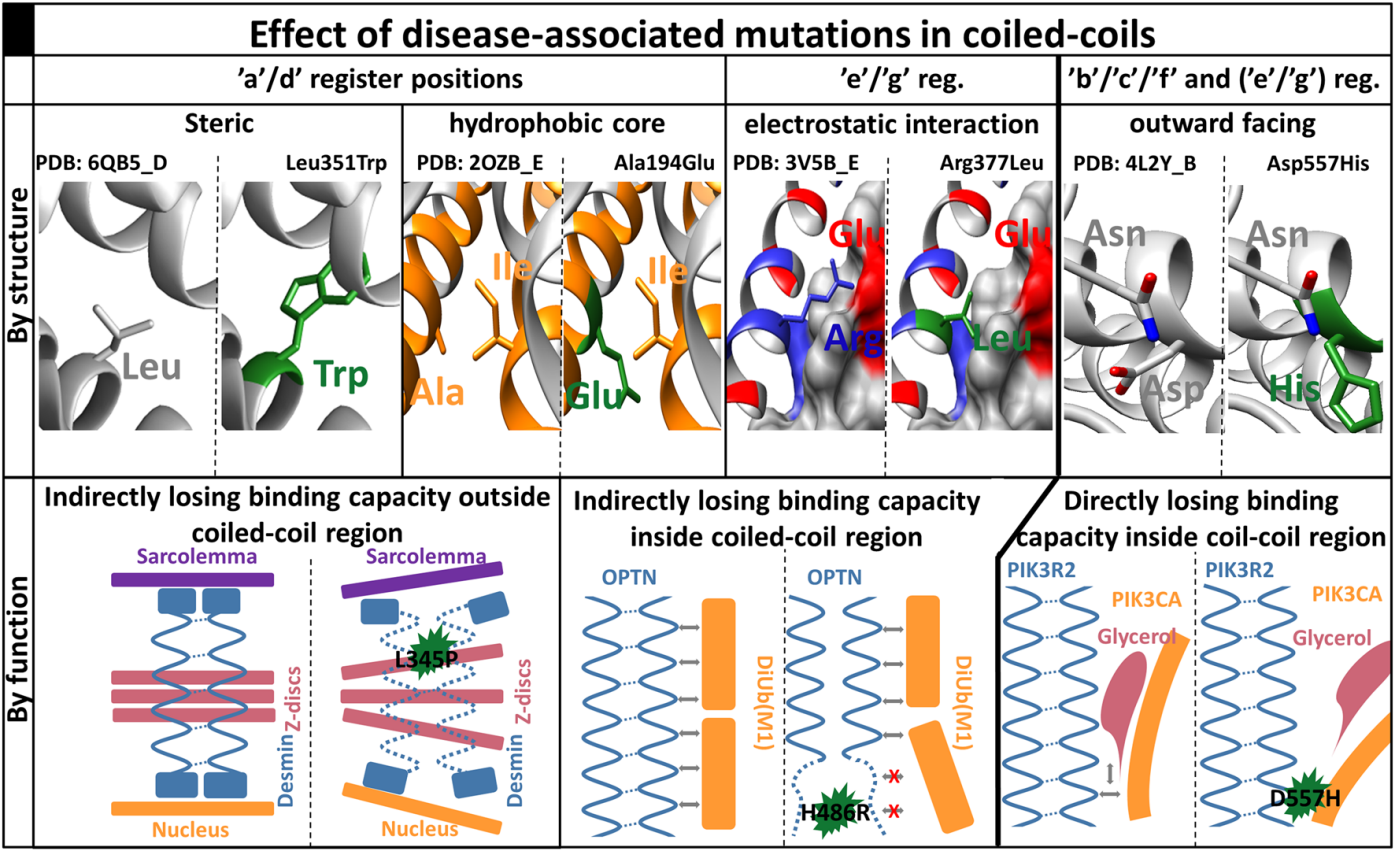
Flavors of disease-causing mutations in coiled-coils. Top row: the structural effect of variations with representative examples how mutations impair coiled-coil structure and function on different registers. From left to right: steric clashes and hydrophobic core disruption on a/d positions; electrostatic change on e/g positions, interaction with other macromolecules on b/c/f positions (note, these mutations on the structure were not relaxed, the ‘Rotamers’ function of Chimera were used to visualize the substitution). Bottom row: functional consequences of mutations. From left to right: damaged coiled-coil loose spacer function and interaction potential outside coiled-coil; mutation on inward facing stabilizing position indirectly influence binding on proximal outward facing residues; mutation on outward facing residue directly abolish the interaction. Left side: wild type structure/function; Right side: perturbed structures/function having pathological condition. Green color indicates disease-causing mutation; On the structure (top row), orange: hydrophobic; blue: positive; red: negative. See the text for more detailed description.

Notably, this effect is strong enough to influence the distribution of variations considering coiled-coils with different lengths. The relative frequency of DMs is significantly higher in shorter coiled-coils (Fig. 2C), as they utilize most of their residues as “N-terminal” segment that may contribute to stability, while in longer coiled-coils, other residues have a lesser role in sustaining the complex form. In contrast, PMs show uniform distribution in coiled-coil regions with different lengths. This effect is also visible on other datasets (Supplementary Fig. 8), confirmed by all prediction methods (Supplementary Fig. 9). It is arguable whether very short predicted coiled-coil segments (below 10 residues) are biologically relevant, however we did not want to tailor prediction outputs. Moreover, omitting the first bin only further strengthens our result.

We performed the same analysis around C-terminal residues, however according to our results the accumulation of DMs is only detectable at the N-terminal region (Supplementary Table 7).

### Oligomerization state affects which register positions are vulnerable

The periodic property of coiled-coils enables a position type classification of residues, grouping amino acid positions based on their location in the helix, uncovering preferred physico-chemical features and interaction types. Regardless of oligomerization state, residues at “a” and “d” positions are often hydrophobic and face each other, forming the core of the complex, while “e” and “g” residues may be charged and promote stability via electrostatic interactions on the outer face of the superhelical structure.

We analyzed the distribution of variations on the different positions, considering different oligomerization states. As expected from early results, PMs are more abundant in every heptad position. However, considering DMs only, residues falling into “stabilizing” positions are more vulnerable to variations (Fig. 3, left). Interestingly, residue type changes affect the heptad positions differently: replacement of amino acids in “a” and “d” positions likely perturb the structure, even when the substitution seems conserving on the basis of physicochemical properties (i.e., variation replacing a hydrophobic residue with another one is also often harmful). In contrast, “e” and “g” positions seem to be slightly more resilient, and residue type change (i.e., charge change) is more often required to disrupt the structure. PMs change the residue type to a lesser extent.

The oligomerization state of the coiled-coil also affects its vulnerability. We have to add, the number of mutations on different datasets highly varies. The less sensitive method (Marcoil) on the less populated dataset (tetramers: ~ 14%) suggests there are 253 disease-associated mutations on this subset (mean 36.14 mutations on each register). The most sensitive method (Ncoils) on the most populated dataset (trimers: ~ 44%) suggests there are 1577 mutations on this subset (with a mean 225.28). Nevertheless, in general, antiparallel formations (both dimers and tetramers) are slightly more preferred targets of DMs. Oligomerization also influences which positions are modulated (Fig. 3, right): “e” and “g” (charged) positions are more often affected by DMs in parallel dimers, while “a” and “d” (hydrophobic) positions are primarily targeted in antiparallel dimers. Hydrophobic interaction promoting positions are less likely to be targeted by DMs in parallel dimers. When these positions are mutated, the mutation changes the type of the residue in almost every case, showing an opposite trend compared to other oligomerization modes.

Variations in trimeric and tetrameric coiled-coils are similar: in these cases, structures are most often perturbed via amino acids in “a”, “g” and “e” positions and also often replace residue type. DMs on “d” positions are rare.

The different prediction methods show high agreement (Supplementary Fig. 10). We also performed the same calculations on the full proteome, and on the random sampled non-redundant dataset (Supplementary Fig. 11), all showing similar results.

In general, there seems to be an opposing trend, that in positions where the DM frequency is lower, any change can carry disease, while in positions where the DM frequency is higher, the mutations more likely change the physico-chemical property of the residue.

### Structure analysis reveals most DMs occur in homooligomeric coiled-coils with a subtle destabilizing effect

To gain detailed insights on how DMs perturb the formation of coiled-coils, we searched for structures in the PDB and identified coiled-coil segments using SOCKET. Although the number of variations falling into characterized coiled-coil structures is rather low, and sometimes insufficient for performing reliable statistical tests to draw convincing conclusions, such analysis can open prospects to recognize interesting trends.

First, we analyzed the distribution of DMs in different heptad positions. As the number of cases was low, we classified the positions into three categories: responsible for hydrophobic stabilization (a, d), electrostatic stabilization (e, g) and outward facing/solvent exposed (b, c, f). Disease-associated mutations are enriched on residues responsible for forming the hydrophobic core of coiled-coils, have nearly the same occurrence as PMs in positions reserved for charged residues, and show decline on outward facing residues (Fig. 4A). Although at first glance this does not seem to confirm prediction data where DMs have a higher frequency on ‘e’ and ‘g’ positions. However, this discrepancy is due to the very different composition of the two datasets with regard to oligomerization state: the most prevalent class of structures are two-stranded antiparallel coiled-coils, the only class where mutations on hydrophobic positions dominate in prediction data too (Fig. 3).

Next, we investigated whether N-terminal segments of the coiled-coils gather more variations. Although both types of variations (PMs and DMs) seem to accumulate in the first seven residues of coiled-coils, the two kinds of variations exhibit an opposing trend, with a higher frequency of DMs around the N-terminal and PMs in other residues (Fig. 4B). Moreover, while PM data is not significant, the distribution of DMs is slightly significant according to the χ^2^ test (*p* < 0.1).

Sequence data alone can be rather difficult to utilize for defining the monomeric or oligomeric state of coiled-coil assemblies, and predictions are also limited in detecting how many strands the coiled-coils are composed of. However, from structural data we can readily classify coiled-coils as monomers (both strands are part of the same protein, typically antiparallel coiled coils with a short linker between the two helices), homooligomers (interaction of identical proteins) or heterooligomers (interaction between different proteins). While PMs show a uniform distribution among these classes, DMs mainly occur in homooligomers (Fig. 4C). The rationale behind this can be that a single mutation might (but in a heterozygous case, not necessarily) affect multiple constituent helices simultaneously, so their effect is instantly multiplied, in contrast to heterooligomers and monomers where the interacting partner/segment does not amplify the impact of the mutation.

The energy change calculated by the introduced mutation can be used as an approximation of the contribution of a mutation to the overall stability of the coiled-coil. Figure 4D shows the calculated energy changes upon mutation in the proteome and in coiled-coil structures. Generally, the mean energetic contribution of PMs can outline the range of changes a protein can tolerate without damage. In both cases (proteome, and coiled-coil proteins), DMs have an average higher ΔΔG. However, in the case of coiled-coils, despite keeping the same trend, both variation types seem to have a lower effect compared to those of other proteins of the proteome.

We also performed the same analyses on the full structure dataset (where the full proteome was assigned to PDB structures). To reduce bias, we removed PDB: 2FXM (Myosin7) from the structures, as 18% of the variations belonged to this protein. On the full dataset, the accumulation of DMs on the first seven residues is visible, yet not significant, furthermore DMs on heterooligomeric proteins are more frequent. Other statistics are in agreement on the full structure dataset (Supplementary Fig. 12, Supplementary Table 20).

Further context can be added by the joint analysis of structural data. Figure 5 shows how DMs are distributed according to their features. Heptad positions with the highest standard deviation corresponding to energetic changes (positions ‘b’, ‘d’, ‘f’) exhibit the most heterogeneous distribution of different types of coiled-coils. Mutations in positions contributing to the hydrophobic core of coiled-coils (‘a’ and ‘d’), as well as the most outward facing residue type (‘f’) available for interactions operate with the most destabilizing energy changes: mutations here likely have more critical effect compared to other positions where there is more (spatial) room for substitutions. Mutations on ‘a’ position more likely affect two-stranded antiparallel coiled-coils (77%; 10 mutations affect two-stranded antiparallel coiled-coils out of total 13 mutations on position ‘a’), which is interestingly not true for the other hydrophobic residue promoting position ‘d’ (22%), also confirmed by the more comprehensive prediction data. Negative (stabilizing) energetic changes are somewhat more likely in homooligomeric coiled-coils (80%), with most cases occurring at position ‘e’. We mapped the variations to only one chain of PDB structures, thus the real energetic contribution of a mutation may be even more stabilizing in homooligomers, abolishing transient interactions. In contrast, most mutations affecting monomeric coiled-coils are definitely highly destabilizing (94%), suggesting greater energetic effect is required to disrupt the overall structure that also includes intrachain interactions outside the coiled-coil, in contrast to mutations of complexes where coiled-coil interchain interactions are the only forces keeping the complex together.

## Discussion

The structural consequences of inherited disease-causing mutations is an often revisited topic^16^. Recently Mohanasundaram et al., investigated how DMs affect coiled-coils^14^. While they mostly focused on pleiotropy and irregularities, in this paper we focused on general patterns. The Mohanasundaram et. al. paper also quantified variations in different PFAM families. In contrast, here we performed an analysis of the non-redundant human proteome. Members of the same family share sequential and structural similarities and might carry out similar functions. For the same reason, these proteins also usually share their mutation hotspots, meaning disease-associated mutations emerge in their same regions. We performed redundancy filtering to rule out bias caused by counting the “same” mutation falling into the same domain regions in more populated families, and de-emphasizing features of smaller protein families. Mohanasundaram et al. also investigated how heptad positions in coiled-coils are affected, however they relied on MarCoil alone. In contrast, we used four different predictors to assess the structural consequences of variations in coiled-coils, then extended our analysis by incorporating structural data and features responsible for the proper assembly of coiled-coil complexes. We found that DMs accumulate in heptad positions critical for the assembly of coiled-coils (in line with the findings published by Mohanasundaram et al.^14^), N-terminal parts of coiled-coils are more abundant in DMs, and mutations mostly affect homooligomeric coiled-coils. Interestingly, in recent analyses some coiled-coil prediction methods showed a rather low accuracy and their result is sometimes contradictory^17,18^, however, based on the agreement of the distribution of variations predicted by different methods, they show balanced performance on our dataset.

### Simple properties of targeted residues suggest how structure is impaired

Sequence properties are often used to characterize substitutions, as they often can be connected to structural changes. In this case, grouping amino acids based on their possible role in coiled-coil formation highlights the critical role of charged residues. While mutations on hydrophobic residues impair coiled-coil structures to the same extent as in the case of globular proteins, charge changes often perturb coiled-coil formation. Notably, not only the change of net charge influences coiled-coils, but residues bearing negative charges also do not seem to be interchangeable. This effect is attributable to the helix formation tendency of glutamic acid^19^ that was also proposed in the case of single-α helices^20^. The most characteristic feature of coiled-coils is their repeated register position pattern. Steric clashes and loss of hydrophobic interactions dominate in ‘a’ and ‘d’ positions (Fig. 6, top, left), while the loss of electrostatic interactions mostly occurs in ‘e’ and ‘g’ positions (Fig. 6, top, middle). Outward-facing residues can also carry essential roles sometimes: they can serve as outside staples that stabilize the alpha-helix by electrostatic interactions, or they can provide a binding site for other molecules (Fig. 6, top, right). For example, the ubiquitin-binding domain (UBAN), conserved in optineurin (OPTN) is part of a coiled-coil, specifically recognizing ubiquitin chains binding to the accessible surface of the coiled-coil^21^. The nuclear factor-κB (NF-κB) pathway plays an important role in regulating inflammation, adaptive and innate immune responses, and cell death via transcriptional targets, such as IL-1β^22^. In the canonical pathway, NF-κB factors are retained in an inactive state via binding to OPTN^21^. The E478G mutation in the UBAN of OPTN abolishes its NF-κB suppressive activity^23^, as residues involved in linear ubiquitin-binding correspond to the residues crucial for keeping NF-κB inactive^24^. The mutations result in significant up-regulation of IL-1β, causing neuroinflammation and neuronal cell death of motor neurons, leading to Amyotrophic Lateral Sclerosis^25^.

### Mutations in coiled-coils influence protein function with different mechanisms

From a functional point of view, mutations falling into distinct structural categories may have different effects. DMs harboring residues contributing to the hydrophobic core usually have an indirect consequence. In the first scenario, the effect of the mutation manifests outside the coiled-coil region. Desmins are large scaffolding proteins connecting the Sarcolemma, Z-discs, and the nucleus^26^. They consist of elongated coiled-coil regions, with a head and tail unit at their termini. Mutations in the coiled-coil regions disrupt the coiled-coil structure (e.g., DESM: L345P), eventually leading to the disorganization of Z discs and affecting the integrity of the cellular IF network^27^ (Fig. 6, bottom, left). Mutations often impair coiled-coils directly, so they lose (some of) their binding affinity to molecules interacting with them. The H486R mutation in OPTN perturbs the structure of the UBAN domain and causes low-grade inflammation that leads to glaucoma^28^. However, in contrast to other mutants that were shown to have a direct role in interacting with ubiquitin, this mutated residue points inside the coiled-coil, and only reduces the binding affinity^29^ (Fig. 6, bottom, middle). In the third scenario, mutations are occurring in the coiled-coil, however on a residue facing outward. An example of disruption of direct binding is the mutation affecting interaction of PIK3CA-PIK3R2-glycerol complex. PIK3R2 possesses a two-stranded coiled-coil and forms a heterodimer regulatory unit with PIK3CA via H-bond between N345 of PIK3CA and D557 of PIK3R2. Their complex structure also preserves a groove, providing a room for binding glycerol^30^, which is perturbed by the D557H mutation. The lost direct contact of Asp sidechain with the glycerol, as well as the lack of negative charge positioning the molecule (which is abolished with the positively charged and larger histidine) were proposed to impact binding negatively^31^. PIK3R2 was associated with Megalencephaly-Polymicrogyria-Polydactyly-Hydrocephalus^32^, although the molecular details of the disease were not revealed yet (Fig. 6, bottom, right). Besides perturbing structural stability and folding leading to toxic conformations, mutations may also modulate degradation or lead to improper trafficking^33^. For example, assembly of the Non-POU domain-containing octamer-binding protein is mediated via antiparallel coiled-coil domains and single-α helices^34,35^. The R293H mutation in the coiled-coil domain was shown to lead to subnuclear mislocalization and resulting in endocrine-related tumors^36^.

### Putative link between N-terminal accumulation of DMs and trigger sequences

The different types of coiled-coils utilizing different strategies to achieve a folded state. Many studies already suggested that highly conserved sequence patterns (so-called trigger sequences) are responsible for initiating coiled-coil assembly: for example a seven residue highly conserved motif is required for the folding of the Human Macrophage Scavenger Receptor oligomerization domain^37^, and germline mutation in this region is associated with prostate cancer risk^38^. Another way to initialize assembly occurs during co-translation, as in the case of Peripherin including two-stranded parallel coiled-coils^39^, which also accomodate a disease-causing mutation at the N-terminal region in one of it's coiled-coil regions^40^. Cotranslational assembly generally occurs via N-terminally biased interaction domains^41^ and a possible interpretation for the N-terminal accumulation of DMs might lie in the co-translational initiation of the folding and stabilization of α-helices as they emerge from the ribosome^42^. Although abolishing the process likely affects superhelix assembly, this phenomenon only serves as an explanation for mutations in parallel coiled-coils. The critical role of terminal regions are also well-marked in antiparallel coiled-coils: SMC1 forms a complex with SMC3 via their globular N- and C-terminal domains. In both proteins the head and tail regions are connected by antiparallel coiled-coils, and most of the identified DMs gather at their beginning/end of the coiled-coil domains^43^. The proposed antiparallel intramolecular coiled-coil of KIF21A gathers several DMs, predominantly occupying the termini of the coiled-coil^44^, responsible for congenital fibrosis. Thus, although the exact molecular background was not revealed yet, there is a substantial amount of evidence supporting the critical role of certain segments in coiled-coils (trigger sites or terminal regions), with an underlied role of N-terminal residues.

## Conclusion

A handful of popular methods are available to predict the effect of variations^45,46^ or to highlight vulnerable regions in proteins^47,48^, yet most of these are based on purely statistical approaches. Methods incorporating structural information are largely limited to general features of PDB structures, or prediction of transmembrane domains or disordered segments, although no currently available methods incorporate features of coiled-coils. We showed that basic properties of coiled-coils, such as register position, oligomerization state and position along the region significantly influence the formation of coiled-coils. Since coiled-coil region prediction typically has short run times, we suggest that including such data into state-of-the-art predictors to increase their accuracy would be feasible.

## Methods

### Datasets

The human proteome was downloaded from UniProt^49^, germline variations were obtained from humsavar^4^ (Supplementary Table 1). For redundancy filtering CD-HIT^50^ was applied on the human proteome in an incremental manner, filtering identical proteins to 90, 70, 50 and finally to 40% identity using 5, 4, 3 and 2 word lengths, respectively (Supplementary Table 2). We performed the analyses on the “non-redundant human proteome”, on the “full human proteome”. Moreover, we also performed “random sampling on the non-redundant dataset”, by selecting 80% of the data 100 times. Differences between the results of various datasets are highlighted in the text.

**Table 1:** Contingency table.

	DM	PM	Residues
Positive	x_1_	x_2_	x_3_
Negative	x_4_	x_5_	x_6_

Positive cases are residues falling into coiled-coil regions/specific positions or proteins containing coiled-coil regions. Negative cases are all other residues/proteins.

### Coiled-coil predictions

Coiled-coil regions were determined using DeepCoil^51^, MarCoil^52^, Ncoils^53^ and Paircoil^54^ (Supplementary Table 3), applying default cutoff values suggested in their descriptive articles. In the case of DeepCoil we utilized the ‘PSSM’ flavor: we generated PSSM for each sequence, using PSI-BLAST with three iterations and 10^−**5**^ e-value cutoff on the SwissProt database. Coiled-coil heptad positions were predicted using MarCoil, Ncoils and Paircoil (Supplementary Table 4). Oligomerization states were defined using LogiCoil (Supplementary Table 4). Single-α Helix regions^55^ were used as a filter, to reduce false positive hits (Supplementary Table 5).

All statistics were calculated independently, using the appropriate predictors—i.e., amino acid substitutions and distribution of variations along the sequence with DeepCoil, MarCoil, Ncoils and Paircoil; impact on heptad positions state by MarCoil, NCoils and PairCoil; distribution in different oligomeric states by LogiCoil (using MarCoil, NCoils and Paircoil as input).

Each time we also calculated the mean value of the results of different predictors—these results are shown in the main text. If there were differences between the results of the applied methods, we noted it in the main text.

### Statistical tests

*χ*^2^ tests were performed in contingency tables (Table 1). Odds ratios were defined as:

$${\text{OR}} = \left( {{\text{x}}_{1} /{\text{x}}_{2} } \right)/\left( {{\text{x}}_{4} /{\text{x}}_{5} } \right)$$

Enrichments on Supplementary Fig. 1 were defined as:

$$\begin{aligned} {\text{Enrichment}}\,\left( {{\text{DMs}}} \right) &= \left( {{\text{x}}_{4} /\left( {{\text{x}}_{4} + {\text{x}}_{1} } \right)} \right)/\left( {{\text{x}}_{6} /\left( {{\text{x}}_{6} + {\text{x}}_{3} } \right)} \right)\\ {\text{Enrichment}}\,\left( {{\text{PMs}}} \right) &= \left( {{\text{x}}_{5} /\left( {{\text{x}}_{5} + {\text{x}}_{2} } \right)} \right)/\left( {{\text{x}}_{6} /\left( {{\text{x}}_{6} + {\text{x}}_{3} } \right)} \right)\end{aligned}$$

*χ*^2^ was applied to find the significance of the relation between DMs and coiled-coils (Supplementary Table 6) and the significant importance of the first seven residues of coiled-coil sequences (Supplementary Table 7).

Kolmogorov–Smirnov tests were used to estimate the significance of the distribution of mutations at the first 28 residues of coiled-coils (Supplementary Table 8) and along the coiled-coil sequences (Supplementary Table 9).

*χ*^2^ test was performed to find the significance of distribution of DMs into coiled-coils with different lengths (Supplementary Table 10).

To estimate the significance of residue changes, we eliminated the sporadic error of the data by performing bootstrap analysis. We randomly selected 80% of the data 100 times and the significance was determined by calculating the average and standard deviations of the data according to the 68-95-99.7 rule (Supplementary Table 11–12).

*χ*^2^ was used to find the significance of the distribution of DMs into different coiled-coils positions (Supplementary Table 13).

All tests and analysis were performed to the different predictors separately. To produce figures, in each case, we calculated the mean of different predictors (Supplementary Table 6–13).

### DiseaseOntology term analysis

Disease ontology terms were mapped using MIM identifiers from humsavar and DiseaseOntology^56^. Only identifiers linked to DMs, where all methods predicted coiled-coil were used. For the analysis the top three level of the ontology was applied, and the number of mutations were counted in each disease category—only terms occurring in coiled-coil containing proteins are shown in Supplementary Table 14, and only terms responsible for at least 5% of all annotated diseases shown in Supplementary Fig. 2. Next we mapped all mutations in a similar manner. Expected values were calculated by normalizing these numbers on each term with the proportion of all coiled-coil mutations.

### Assigning structures to amino acid sequences

We used BLAST on sequences from the non-redundant human proteome against the PDB with 10^−**5**^ e-value. Chimeric proteins were discarded. We used the greedy algorithm to select structures with 100% identity, with the most variations mapped on them (Supplementary Table 15). On all PDB structures we considered biomatrix transformations as defined in the PDB files to detect all possible coiled-coils.

### Calculating structural and energetic properties

We detected coiled-coils using SOCKET^57^ with default settings. Coiled-coil features [heptad positions, the number of strands, angle of strands (Supplementary Table 15–16)] were determined based on SOCKET output. For monomer/homooligomer/heterooligomer assignment, we checked which BLAST query corresponds to the detected coiled-coil regions (Supplementary Table 18). Energy calculations were performed using FoldX^58^. ΔΔ*G* calculations were executed on previously optimized structures and were performed five times. All reported ΔΔ*G* values represent the average of these independent runs. In 76 cases (less than 1%) we experienced problems with FoldX, these cases were omitted (Supplementary Table 19). Calculated structural features shown on Fig. 4 are based on values from Supplementary Table 20. Energetic changes on figure are categorized as highly stabilizing (< −1.84 kcal/mol), stabilizing (− 1.84 to − 0.92 kcal/mol), slightly stabilizing (− 0.92 to − 0.46 kcal/mol), neutral (− 0.46 to + 0.46 kcal/mol), slightly destabilizing (+ 0.46 to + 0.92 kcal/mol), destabilizing (+ 0.92 to + 1.84 kcal/mol) and highly destabilizing (> +1.84 kcal/mol).

### Visualization

Images were prepared using UCSF Chimera^59^.

## Supplementary information

Supplementary information.

Supplementary Tables.

## References

[1] Dong G, Medkova M, Novick P, Reinisch KM (2007). A catalytic coiled coil: Structural insights into the activation of the Rab GTPase Sec4p by Sec2p. Mol. Cell.

[2] Truebestein L, Leonard TA (2016). Coiled-coils: The long and short of it. BioEssays.

[3] Hayashi M (2010). The postsynaptic density proteins homer and shank form a polymeric network structure. Neurosci. Res..

[4] Crick FHC (1953). The packing of α-helices: Simple coiled-coils. Acta Crystallogr. A.

[5] Burkhard P, Stetefeld J, Strelkov SV (2001). Coiled coils: A highly versatile protein folding motif. Trends Cell Biol..

[6] Moutevelis E, Woolfson DN (2009). A periodic table of coiled-coil protein structures. J. Mol. Biol..

[7] Lupas AN, Bassler J, Dunin-Horkawicz S (2017). The structure and topology of α-helical coiled coils. Subcell. Biochem..

[8] Mason JM, Arndt KM (2004). Coiled coil domains: Stability, specificity, and biological implications. ChemBioChem.

[9] Hicks MR, Holberton DV, Kowalczyk C, Woolfson DN (1997). Coiled-coil assembly by peptides with non-heptad sequence motifs. Fold. Des..

[10] Kammerer RA (1998). An autonomous folding unit mediates the assembly of two-stranded coiled coils. Proc. Natl. Acad. Sci. USA.

[11] Ng DP, Poulsen BE, Deber CM (2012). Membrane protein misassembly in disease. Biochim. Biophys. Acta.

[12] Pajkos M, Mészáros B, Simon I, Dosztányi Z (2012). Is there a biological cost of protein disorder? Analysis of cancer-associated mutations. Mol. BioSyst..

[13] Gao M, Zhou H, Skolnick J (2015). Insights into disease-associated mutations in the human proteome through protein structural analysis. Structure.

[14] Mohanasundaram KA, Grover MP, Crowley TM, Goscinski A, Wouters MA (2017). Mapping genotype-phenotype associations of nsSNPs in coiled-coil oligomerization domains of the human proteome. Hum. Mutat..

[15] Woolfson DN (2017). Coiled-coil design: Updated and upgraded. Subcell. Biochem..

[16] Dobson L, Mészáros B, Tusnády GE (2018). Structural principles governing disease-causing germline mutations. J. Mol. Biol..

[17] Simm D, Hatje K, Waack S, Kollmar M (2020). Protein function prediction in genomes: Critical assessment of coiled-coil predictions based on protein structure data. bioRxiv.

[18] Szappanos B, Süveges D, Nyitray L, Perczel A, Gáspári Z (2010). Folded-unfolded cross-predictions and protein evolution: The case study of coiled-coils. FEBS Lett..

[19] Chou PY, Fasman GD (1978). Prediction of the secondary structure of proteins from their amino acid sequence. Adv. Enzymol. Relat. Areas Mol. Biol..

[20] Süveges D, Gáspári Z, Tóth G, Nyitray L (2009). Charged single alpha-helix: a versatile protein structural motif. Proteins.

[21] Rahighi S (2009). Specific recognition of linear ubiquitin chains by NEMO is important for NF-kappaB activation. Cell.

[22] Cogswell JP (1994). NF-kappa B regulates IL-1 beta transcription through a consensus NF-kappa B binding site and a nonconsensus CRE-like site. J. Immunol..

[23] Li F (2016). Structural insights into the interaction and disease mechanism of neurodegenerative disease-associated optineurin and TBK1 proteins. Nat. Commun..

[24] Nakazawa S (2016). Linear ubiquitination is involved in the pathogenesis of optineurin-associated amyotrophic lateral sclerosis. Nat. Commun..

[25] Liu Z (2018). ALS-associated E478G mutation in human OPTN (Optineurin) promotes inflammation and induces neuronal cell death. Front. Immunol..

[26] Hnia K, Ramspacher C, Vermot J, Laporte J (2015). Desmin in muscle and associated diseases: Beyond the structural function. Cell Tissue Res..

[27] Goldfarb LG, Dalakas MC (2009). Tragedy in a heartbeat: Malfunctioning desmin causes skeletal and cardiac muscle disease. J. Clin. Invest..

[28] Toth RP, Atkin JD (2018). Dysfunction of optineurin in amyotrophic lateral sclerosis and glaucoma. Front. Immunol..

[29] Li F (2018). Structural insights into the ubiquitin recognition by OPTN (optineurin) and its regulation by TBK1-mediated phosphorylation. Autophagy.

[30] Zhao Y (2014). Crystal structures of PI3Kα complexed with PI103 and its derivatives: New directions for inhibitors design. ACS Med. Chem. Lett..

[31] Terrone G (2016). De novo PIK3R2 variant causes polymicrogyria, corpus callosum hyperplasia and focal cortical dysplasia. Eur. J. Hum. Genet..

[32] Mirzaa GM (2015). Characterisation of mutations of the phosphoinositide-3-kinase regulatory subunit, PIK3R2, in perisylvian polymicrogyria: A next-generation sequencing study. Lancet Neurol..

[33] Thomas PJ, Qu B-H, Pedersen PL (1995). Defective protein folding as a basis of human disease. Trends Biochem. Sci..

[34] Passon DM (2012). Structure of the heterodimer of human NONO and paraspeckle protein component 1 and analysis of its role in subnuclear body formation. Proc. Natl. Acad. Sci. USA.

[35] Dobson L, Nyitray L, Gáspári Z (2015). A conserved charged single α-helix with a putative steric role in paraspeckle formation. RNA.

[36] Kharade SS, Parekh VI, Agarwal SK (2018). Functional defects from endocrine disease-associated mutations in HLXB9 and its interacting partner. NONO. Endocrinol..

[37] Frank S, Lustig A, Schulthess T, Engel J, Kammerer RA (2000). A distinct seven-residue trigger sequence is indispensable for proper coiled-coil formation of the human macrophage scavenger receptor oligomerization domain. J. Biol. Chem..

[38] Xu J (2002). Germline mutations and sequence variants of the macrophage scavenger receptor 1 gene are associated with prostate cancer risk. Nat. Genet..

[39] Chang L, Shav-Tal Y, Trcek T, Singer RH, Goldman RD (2006). Assembling an intermediate filament network by dynamic cotranslation. J. Cell Biol..

[40] Kohl S (1997). RDS/peripherin gene mutations are frequent causes of central retinal dystrophies. J. Med. Genet..

[41] Schwarz A, Beck M (2019). The benefits of cotranslational assembly: A structural perspective. Trends Cell Biol..

[42] Kramer G, Boehringer D, Ban N, Bukau B (2009). The ribosome as a platform for co-translational processing, folding and targeting of newly synthesized proteins. Nat. Struct. Mol. Biol..

[43] Deardorff MA (2007). Mutations in cohesin complex members SMC3 and SMC1A cause a mild variant of cornelia de Lange syndrome with predominant mental retardation. Am. J. Hum. Genet..

[44] Bianchi S (2016). Structural basis for misregulation of kinesin KIF21A autoinhibition by CFEOM1 disease mutations. Sci. Rep..

[45] Adzhubei I, Jordan DM, Sunyaev SR (2013). Predicting functional effect of human missense mutations using PolyPhen-2. Curr. Protoc. Hum. Genet..

[46] Kulandaisamy A, Zaucha J, Sakthivel R, Frishman D, MichaelGromiha M (2020). Pred-MutHTP: Prediction of disease-causing and neutral mutations in human transmembrane proteins. Human Mutat..

[47] Mészáros B, Zeke A, Reményi A, Simon I, Dosztányi Z (2016). Systematic analysis of somatic mutations driving cancer: Uncovering functional protein regions in disease development. Biol. Direct.

[48] Niu B (2016). Protein-structure-guided discovery of functional mutations across 19 cancer types. Nat. Genet..

[49] UniProt Consortium (2019). UniProt: A worldwide hub of protein knowledge. Nucleic Acids Res..

[50] Huang Y, Niu B, Gao Y, Fu L, Li W (2010). CD-HIT Suite: A web server for clustering and comparing biological sequences. Bioinformatics.

[51] Ludwiczak J, Winski A, Szczepaniak K, Alva V, Dunin-Horkawicz S (2019). DeepCoil-a fast and accurate prediction of coiled-coil domains in protein sequences. Bioinformatics.

[52] Delorenzi M, Speed T (2002). An HMM model for coiled-coil domains and a comparison with PSSM-based predictions. Bioinformatics.

[53] Lupas A, Van Dyke M, Stock J (1991). Predicting coiled coils from protein sequences. Science.

[54] McDonnell AV, Jiang T, Keating AE, Berger B (2006). Paircoil2: Improved prediction of coiled coils from sequence. Bioinformatics.

[55] Dudola D, Tóth G, Nyitray L, Gáspári Z (2017). Consensus prediction of charged single alpha-helices with CSAHserver. Methods Mol. Biol..

[56] Schriml LM (2012). Disease Ontology: A backbone for disease semantic integration. Nucleic Acids Res..

[57] Walshaw J, Woolfson DN (2001). Socket: A program for identifying and analysing coiled-coil motifs within protein structures. J. Mol. Biol..

[58] Delgado J, Radusky LG, Cianferoni D, Serrano L (2019). FoldX 5.0: Working with RNA, small molecules and a new graphical interface. Bioinformatics.

[59] Pettersen EF (2004). UCSF Chimera—A visualization system for exploratory research and analysis. J. Comput. Chem..

