# Supplementary material for: Distribution of disease-causing germline mutations in coiled-coils implies an important role of their N-terminal region
Source: Sci Rep. 2020 Oct 15;10:17333. doi: 10.1038/s41598-020-74354-9 (PMC7562717; doi:10.1038/s41598-020-74354-9)
Supplement: Supplementary file 1 — Supplementary information. [file 41598_2020_74354_MOESM1_ESM.docx]

Distribution of disease-causing germline mutations in coiled-coils implies an important role of their N-terminal region

**Zsofia E. Kalman^1,2^, Bálint Mészáros^3^, Zoltán Gáspári^1,*^, Laszlo Dobson^1,4*^**

^1^Faculty of Information Technology and Bionics, Pázmány Péter Catholic University, Práter u. 50/A, 1083 Budapest, Hungary

^2^3in-PPCU Research Group, 2500 Esztergom, Hungary

^3^Structural and Computational Biology Unit, European Molecular Biology Laboratory, Meyerhofstraße 1, 69117 Heidelberg, Germany

^4^Research Centre for Natural Sciences, Magyar Tudósok Körútja 2, 1117 Budapest, Hungary

*To whom correspondence should be addressed: [gaspari.zoltan@itk.ppke.hu](mailto:dobson.laszlo.imre@itk.ppke.hu), [dobson.laszlo@ttk.mta.hu](mailto:dobson.laszlo.imre@itk.ppke.hu)

# Supplementary Results

## DMs primarily target coiled-coil segment containing proteins outside the coiled-coil region

In general, PMs have a higher overall abundance in the human proteome than DMs, as observed in many previous studies. At the protein level, there is a slight increase in the frequency of DMs in coiled-coil containing proteins compared to other proteins in the proteome, yielding an odds ratio of 1.03 against PMs (Supplementary Figure 1).Notably, prediction methods exhibit some inconsistency regarding these statistics: DeepCoil predicts less coiled- coils and produces a negative odds ratio, while the other three methods show the inverse trend. Surprisingly in the light of the above observation, coiled-coil regions are depleted in DMs compared to non-coiled-coil residues, agreed by all prediction methods (odds ratio: 0.56). A possible explanation for this phenomenon might be that coiled-coils are generally less vulnerable to harmful variations. However, these proteins carry essential functions as they are more frequently occupied by DMs.

## Impaired coiled-coils are mostly associated with central nervous system diseases

To get a better picture of what types of diseases emerge from the analyzed variations, we used DiseaseOntology to group diseases into categories based on their MIM identifier. Such ontologies often have some level of annotation bias, as the included proteins are not necessarily annotated to the same extent. To avoid drawing false conclusions we did not calculate p-values, and only counted the number of occurrences (and also highlighted the expected number of occurrences for comparison, Supplementary Figure 2). shows the most commonly occurring disease classes, where a DM directly disrupts the structure of a coiled-coil. According to our analysis, the most enriched disease terms are skin diseases, muscular diseases, carbohydrate metabolic diseases, and central nervous system diseases. Coiled-coil DMs are also enriched in more general terms, such as integumentary system diseases, musculoskeletal system diseases, and nervous system diseases. There is also a subtle abundance of conditions related to metabolic diseases that can be acquired according to the annotation.

# Supplementary Figures

***
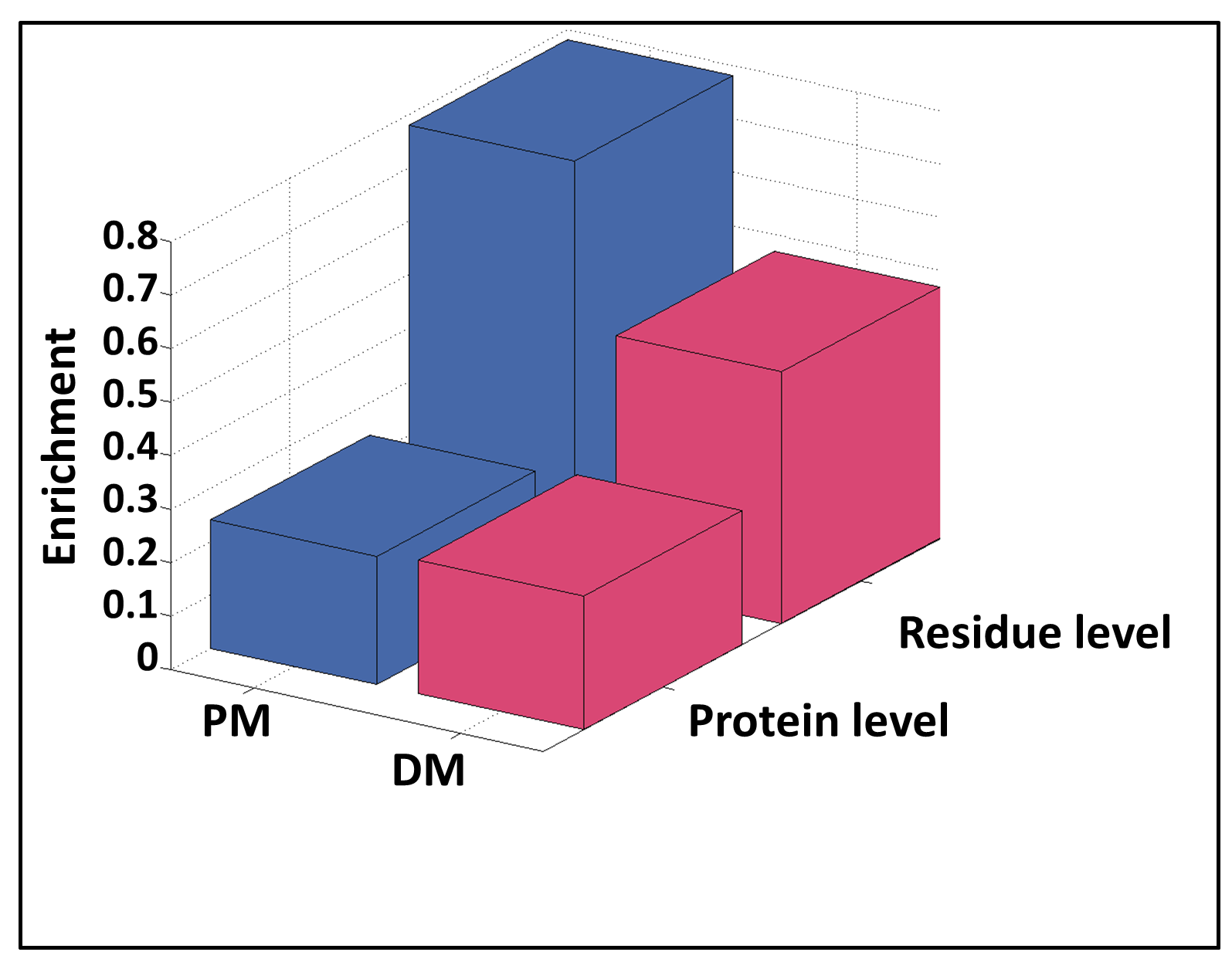
***

***SFigure 1: Relation between DMs and coiled-coils.*** *Enrichment (the ratio of the relative frequency of coiled-coils and non-coiled-coils) were calculated at residue level (coiled-coil residues) and protein level (coiled-coil containing proteins). PMs: blue; DMs: red*

***SFigure 2: Coiled-coil regions with multiple mutations.*** *Ratio of coiled-coil regions with one or more mutations.*


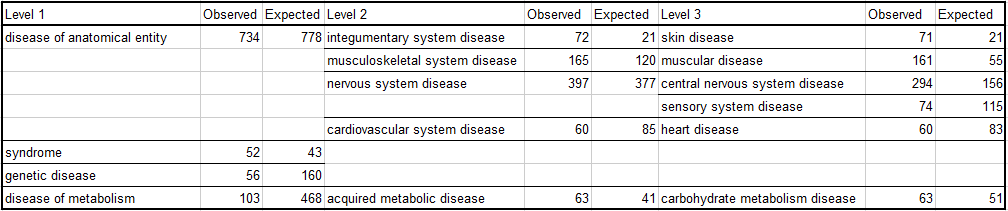


***SFigure 3: DiseaseOntology term analysis.*** *Only terms responsible for at least 5% of all annotated diseases are shown in the top 3 levels of the ontology. N is the number of mutations disrupting coiled-coil structures (in parenthesis expected values are shown).*


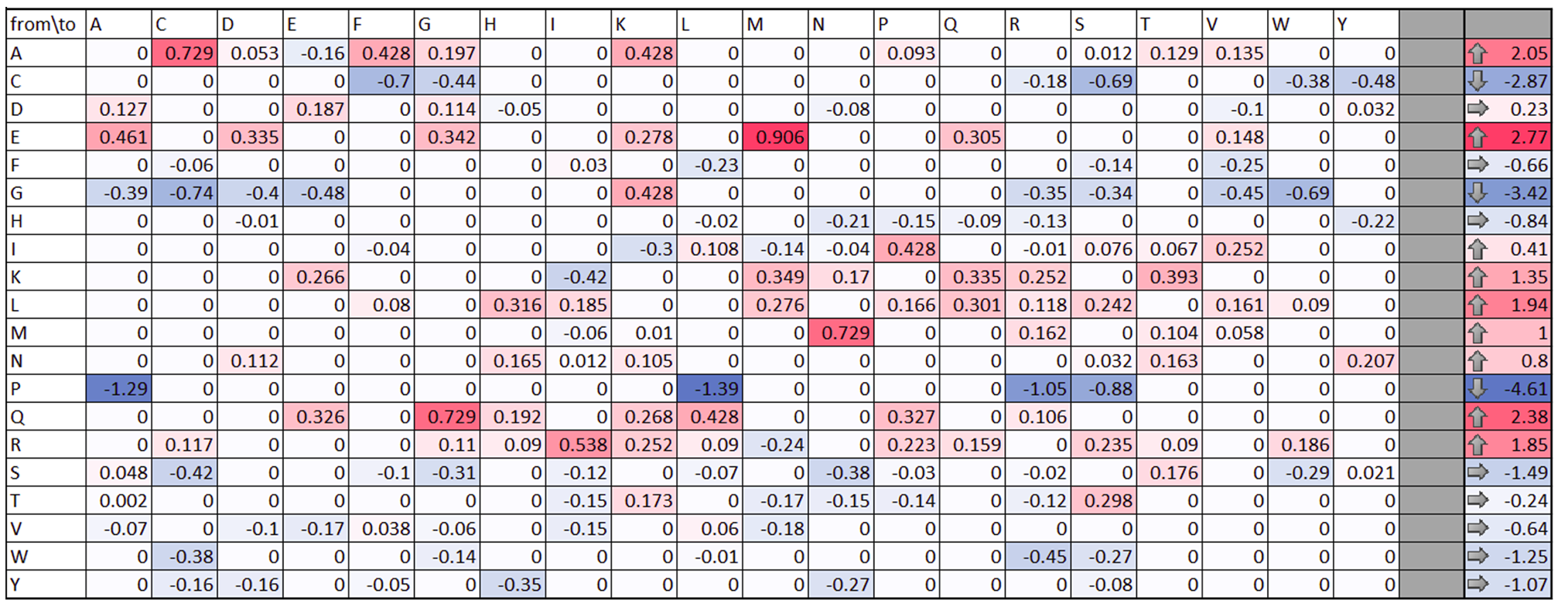


***SFigure 4: Amino acid changes in coiled-coils.*** *Left) Residue change preferences by DMs in the proteome (negative values, also marked with the shades of blue) and in coiled-coil regions (positive values, also marked with the shades of red). Values show the logarithm of ratio of DMs changing given residues types Right) Targeted residue type preferences by DMs in the proteome (negative values) and in coiled-coil regions (positive values).*

*
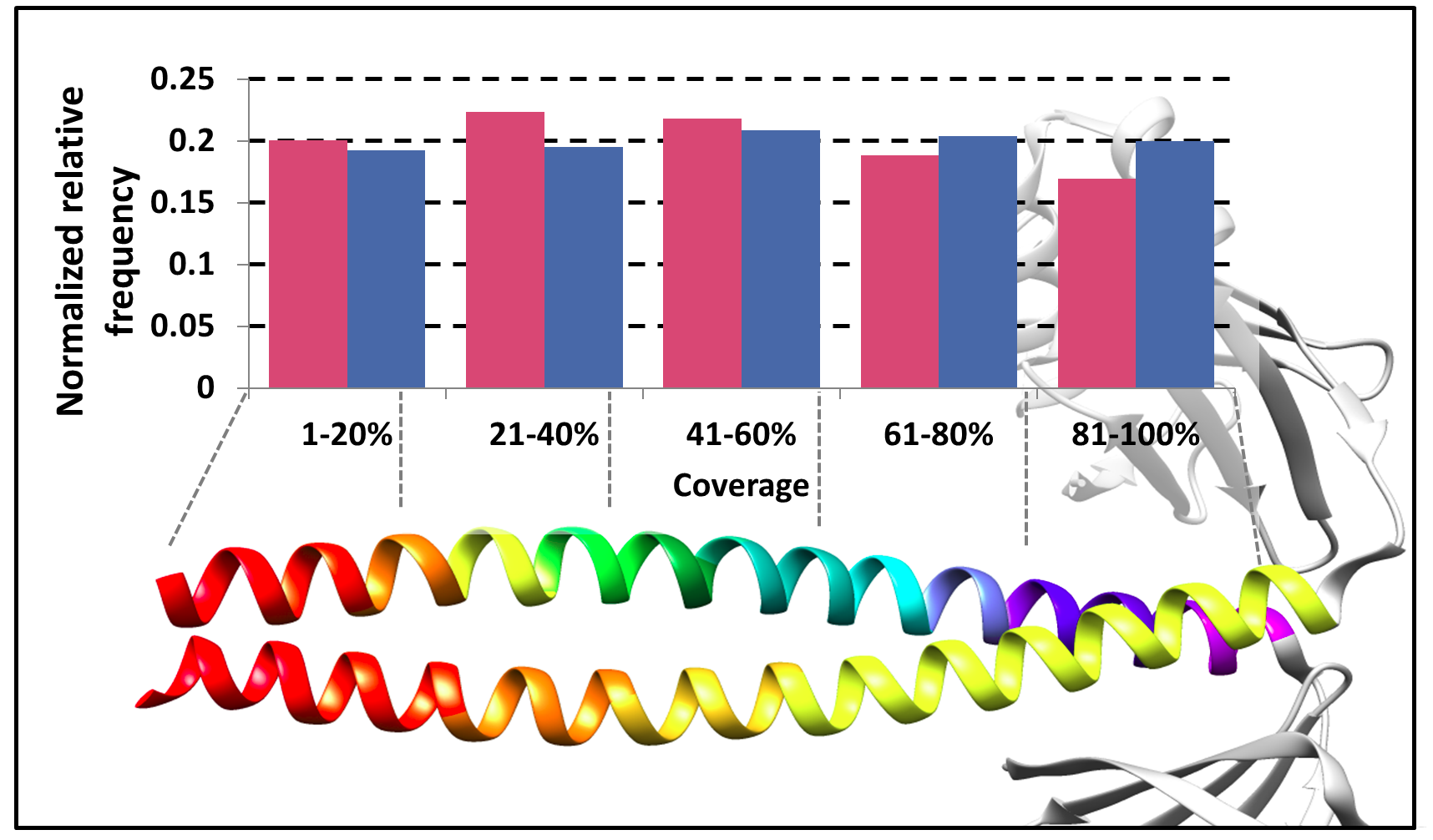
*

***SFigure 5: Variations along coiled-coil segments.*** *Distribution of variations in the sequence. X-axis shows the coverage of the sequences.*


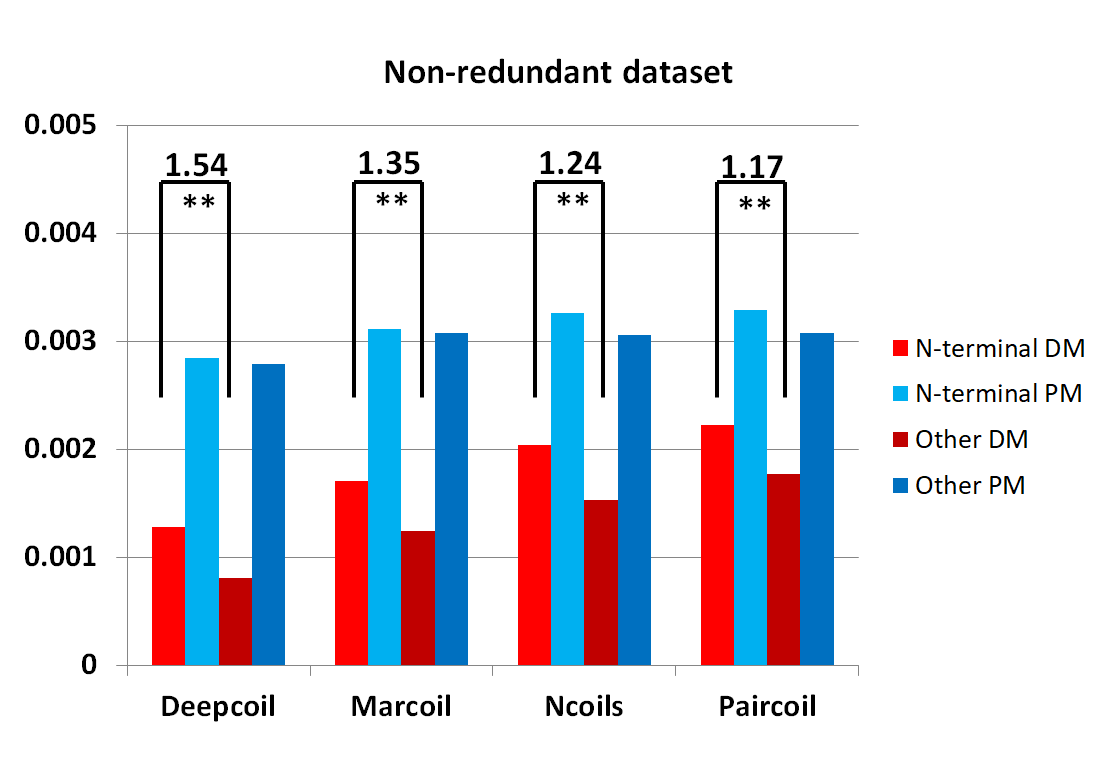


***SFigure 6:*** *Relative frequency of coiled-coil residues targeted at N-terminal of the coiled-coil, and other coiled-coil residues. Results were calculated on the non-redundant dataset using the methods idependently.*

***SFigure 7:*** *Relative frequency of coiled-coil residues targeted at N-terminal of the coiled-coil, and other coiled-coil residues. Results were calculated from the mean of different predictors on the non-redundant dataset, on the non-redundent dataset using random sampling and on the full proteome.*

**Sfigure 8:** *Distributions of variations in coiled-coils with different lengths on different datasets. Results were calculated from the mean of different predictors.*

**Sfigure 9:** *Distributions of variations in coiled-coils with different lengths. Results were calculated independently by different predictors.*


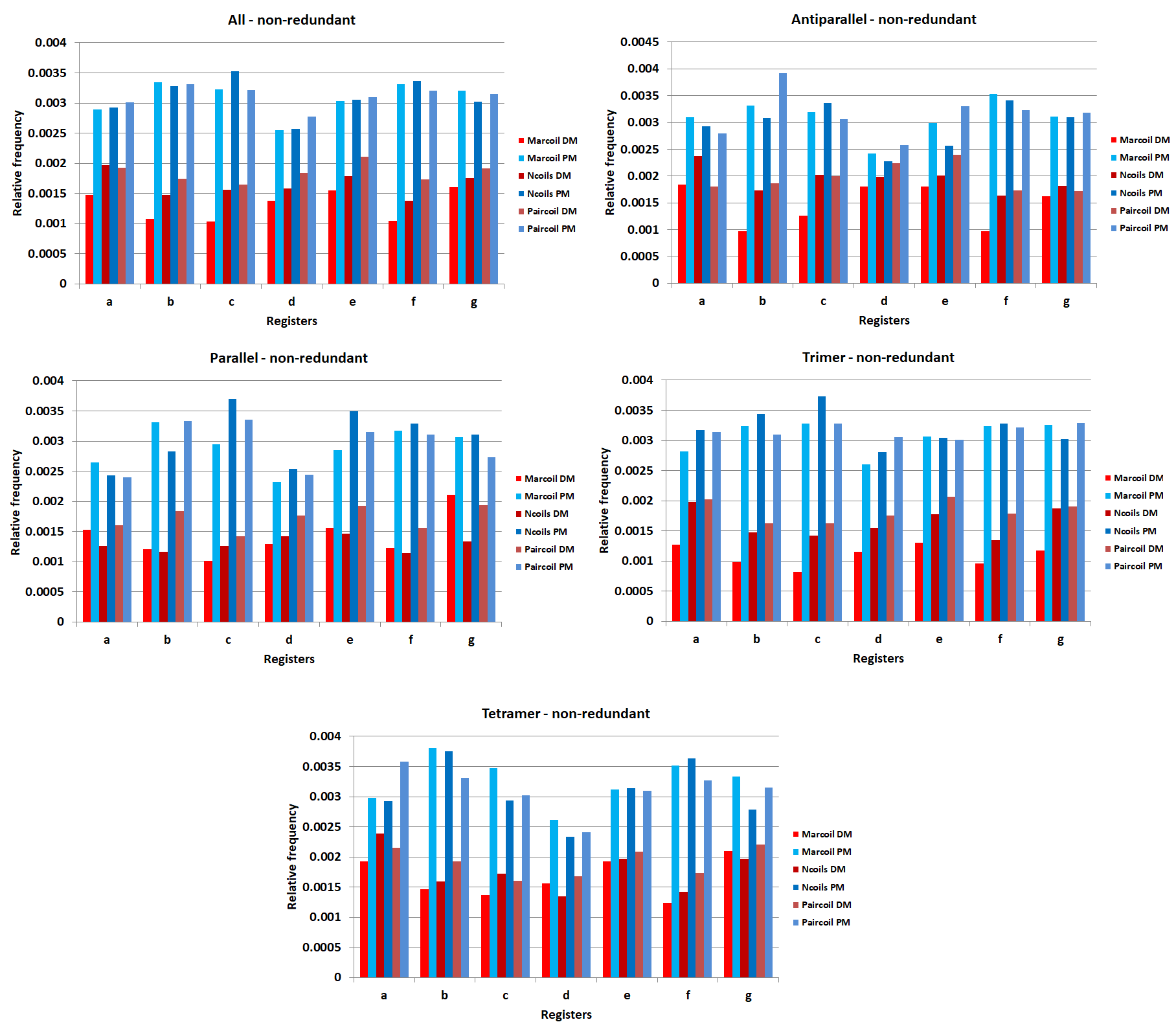


**Sfigure 10:** *Distributions of variations in different heptad posotions. Results were calculated independently by different predictors.*


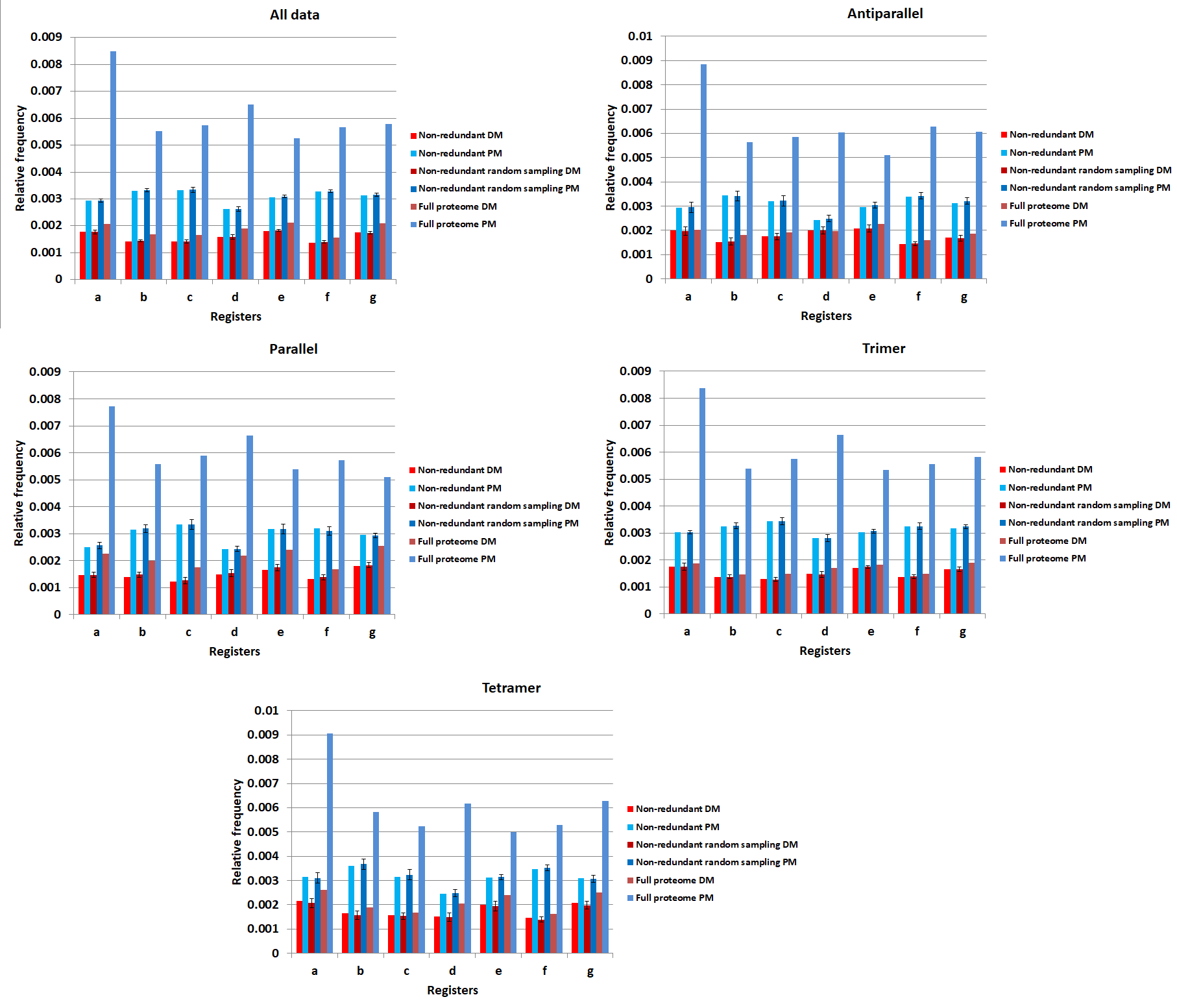


**Sfigure 11:** *Distributions of variations in different heptad posotions. Results were calculated on each dataset, and the mean value of different methods are shown.*


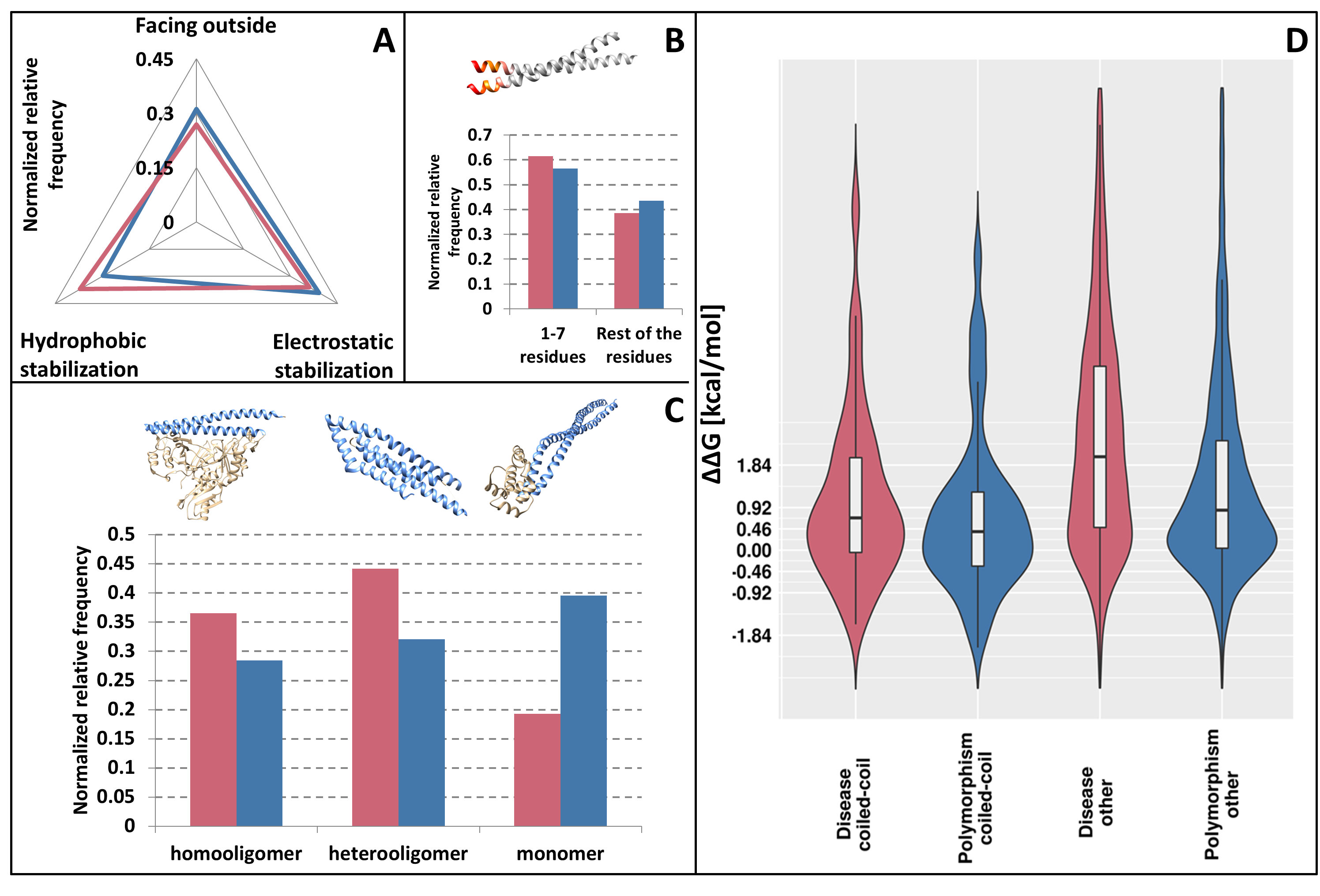


***Figure 12: Distribution of variation on all human PDB structures.*** *A) Distribution of variations based on the register position types. B) Distribution of variations in the N-terminal seven residues and in other segments of coiled-coils. C) Distribution of variations according to the oligomerization state of structures. D) Energy change distributions in coiled-coils (right) and in other proteins from the human proteome (left). Red: DMs; blue: PMs.*
